# Anxiety, depression, and sleep disturbances among people on long-term efavirenz-based treatment for HIV: a cross-sectional study in Beijing, China

**DOI:** 10.1186/s12888-022-04366-4

**Published:** 2022-11-16

**Authors:** Jing Xiao, Ying Liu, Bei Li, Leidan Zhang, Junyan Han, Hongxin Zhao

**Affiliations:** 1 Peking University Ditan Teaching Hospital, Beijing, 100015 China; 2 National Center for Infectious Diseases, Beijing Ditan Hospital, Capital Medical University, Beijing, 100015 China; 3 Clinical and Research Center of Infectious Diseases, Beijing Ditan Hospital, Capital Medical University, Beijing, 100015 China; 4 Beijing Key Laboratory of Emerging Infectious Diseases, Institute of Infectious Diseases, Beijing Ditan Hospital, Capital Medical University, Beijing, 100015 China; 5 Beijing Institute of Infectious Diseases, Beijing, 100015 China

**Keywords:** Antiretroviral therapy, Neuropsychiatric adverse effect, Anxiety, Depression, Sleep disturbances

## Abstract

**Background:**

Efavirenz (EFV)-induced neuropsychiatric toxicity bothers people living with HIV (PLHIV). Neuropsychiatric adverse effects of EFV may differ by length of time on EFV-based antiretroviral treatment (ART).

**Methods:**

A cross-sectional, single-center study was conducted at Beijing Ditan Hospital in China from June–August 2020 among ART-experienced PLHIV who were on long-term EFV-based ART. 424 eligible virological suppressed participants were enrolled and divided into four groups according to time on EFV-based ART: group A (0.5 ≤ ART < 2 year), B (2 ≤ ART < 4 year), C (4 ≤ ART < 6 year), and D (ART ≥ 6 year). The questionnaires about 12-item Short Form Health Survey (SF-12), Hospital Anxiety and Depression Scale (HADS) and Pittsburgh Sleep Quality Index (PSQI) were administered to assess neuropsychiatric adverse events of EFV among different groups.

**Results:**

Overall mental component summary scores (MCS) of SF-12 in PLHIV was 50.2, which was lower than general population. Overall prevalence of anxiety, depression and sleep disturbances was 15.6%, 15.3% and 58%, respectively. Prevalence of anxiety, depression and sleep disturbances did not vary significantly between the time-on-ART groups. Anxiety, depression, sleep disturbances had no correlation with time on EFV-based ART or CD4^+^ T cells counts.

**Conclusions:**

In ART-experienced PLHIV in China, neuropsychiatric adverse events exist persistently and prevalence do not significantly change with prolonged time on EFV-based ART. The prevalence of sleep disturbances was high, suggesting that clinicians should pay more attention to long-standing psychiatric health to perform early and effective interventions.

**Supplementary Information:**

The online version contains supplementary material available at 10.1186/s12888-022-04366-4.

## Introduction

The three leading causes of burden of disease in 2030 are projected to include HIV/AIDS, unipolar depressive disorders, and ischaemic heart disease [1], and mental disorders have been leading causes of disability worldwide [2]. Due to the particularities of AIDS, people living with HIV (PLHIV) face more challenges than HIV-uninfected individuals, including a loss of social support, mental health issues, discrimination and cognitive impairment [3]. More attention should be brought to the neuropsychiatric disturbances in PLHIV. Researchers have proposed adding a fourth 90 to the testing and treatment target: ensure that 90% of PLHIV with viral load suppression have a good health-related quality of life [4].

Neuropsychiatric problems such as anxiety, depression and insomnia are prevalent among PLHIV worldwide and prevalence of anxiety, poor sleep quality is 19–37% [5], 52.5% [6]in previously published literature, respectively. During the COVID-19 outbreak in China, prevalence of depression is 21.1% in the general population [7], in stark contrast, a meta-analysis in China among PLHIV estimated the prevalence of having depression as 54% [8], which is up to seven-fold higher than the general population. These disorders often diminish quality of life, and result in low patient adherence or even suicidal ideation [6, 9]. The reasons underlying neuropsychiatric problems in PLHIV may include HIV neurotoxicity, recreational drugs alter brain hemostasis and subsequently damage the CNS, side effects of antiretroviral therapy (ART), HIV stigmatization and social factors [10–12]. Despite its widespread use, efavirenz (EFV) has been linked to neuropsychiatric adverse events with a related neuropsychiatric adverse events rate among PLHIV as high as 25–70% [13], evidence from cross-sectional studies buttress that the risk increases by 1.6-fold for being diagnosed with an anxiety disorder, or mixed anxiety and depression among patients receiving EFV-containing therapy in Thailand [14].

China implemented the national AIDS control policy “Four Frees and One Care” in 2003 and the National Free Antiretroviral Treatment Program (NFATP) established concurrently. The original NFATP first-line regimen was zidovudine (AZT) + didanosine (ddI) + nevirapine (NVP) or stavudine (d4T) + ddI + NVP. EFV was added subsequently to the list of NFATP-sponsored free antiretrovirals in 2005 and combination TDF + 3TC + EFV was newly adopted as first-line therapy in the 2011 Chinese National Guidelines for HIV/AIDS Diagnosis [15, 16]. Dolutegravir (DTG) has been recommended as the preferred first-line ART for all PLHIV by WHO [17], however most patients in China can’t afford DTG and EFV still remains one of the most widely used ART drugs in China, partly because of limited drug accessibility.

EFV-based antiretroviral regimens were the mainstream regimen in China [18], though the introduction of EFV decreased the prevalence of Nevirapine (NVP)-induced liver toxicity and allergy, EFV-induced neuropsychiatric toxicity bothers patients, thus EFV-containing regimens are selected as alternative regimen as determined by DHHS (Department of Health and Human Services), IAS (International AIDS Society), European AIDS Clinical Society, WHO (World Health Organization). Entering the era of integrase strand transfer inhibitor (INSTI), when considering whether EFV still suits as one of the mainstream substances in ART regimen in China, it is important to be aware of its neuropsychiatric adverse effect to PLHIV.

In a prospective observational study at Youan Hospital in Beijing, researchers found PLHIV with neuropsychiatric adverse effect at baseline would have severe neuropsychiatric disorders over the 12 months [19, 20], however another prospective trial in Peking Union Medical College Hospital found PLHIV diagnosed with depression had no progression in neuropsychiatric co-morbidities [21]. At present, the specific effects of EFV on neuropsychiatric conditions among PLHIV is still controversial, furthermore those studies have short observation periods considering that PLHIV with ART need lifelong medication. Half of the patients discontinued EFV later than 12 months after initiation of treatment and this suggested neuropsychiatric toxicity persisted longer [22]. Further research with a longer observational time is thus necessary. To help address knowledge gaps, we used a cross-sectional design to make comparisons of prevalence of neuropsychiatric adverse events among PLHIV with different time on EFV-based ART for up to 15 years in China.

## Methods

### Study design and participants

This study is a cross-sectional, single-center study, and the clinical data of 424 ART-experienced PLHIV is based on the research project “Efficacy and safety of Efavirenz 400 mg-based regimens switching from 600 mg-based regimens with virologic suppression in HIV-1 infected patients: a randomized, open-label, non-inferiority study” from Capital’s Funds for Health Improvement and Research (No. 2020–2-2174). HIV-infected patients treated at Beijing Ditan Hospital, Capital Medical University from June to August 2020 in China were screened for study eligibility. Patients were eligible if they were PLHIV older than 18 years, and previously initiated first-line ART with an EFV (600 mg)-based regimen and virologically suppressed (HIV-RNA < 50 copies/ml) for at least 6 months prior to enrollment. Participants were excluded if they were pregnant or planning to get pregnant, had tuberculous coinfection, or had laboratory values outside predefined ranges (aspartate aminotransferase [AST], or alanine aminotransferase [ALT], or both > 5 times the upper limit of normal, bilirubin level more than 2.5 times the upper limit of the normal range, and serum creatinine level in excess of 1.5 times the upper limit of the normal range). The clinicians informed the eligible patient of the detailed study objectives, the study process, and the potential benefits and risks and then patients signed informed consent. 424 participants all provided written informed consent for treatment prior to enrollment. Signed informed consent as well as questionnaire were stored in a locked cabinet. Each eligible participants in our study got a computer-generated randomization number, and their data was anonymized and only used for the purposes of the study. Periodical laboratory tests including routine blood tests, routine urine tests, liver function tests, renal function test and blood lipids were provided free of charge. The study protocol was approved by the Ethics Committee of Beijing Ditan Hospital, Capital Medical University (identification number: 2020–029-02).

**Fig. 1:**
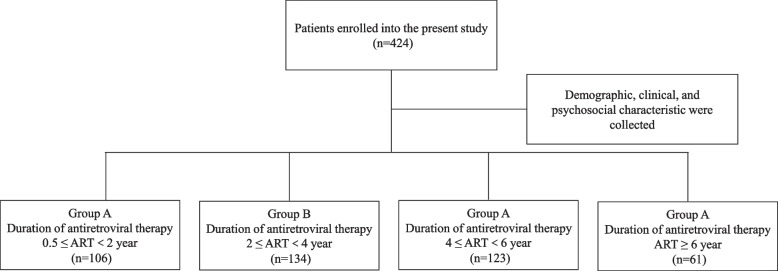
Study design flowchart. According to time on EFV-based ART, 424 participants were divided into 4 groups: group A (0.5 ≤ ART < 2 years), group B (2 ≤ ART < 4 years), group C (4 ≤ ART < 6 years), and group D (ART ≥ 6 years). Abbreviations: *EFV, efavirenz; ART, antiretroviral therapy*

We confirmed when patients initiated EFV-based ART by querying a hospital-based electronic medical record system. According to time on EFV-based ART, 424 participants were divided into 4 groups: group A (0.5 ≤ ART < 2 years), group B (2 ≤ ART < 4 years), group C (4 ≤ ART < 6 years), and group D (ART ≥ 6 years) (Fig. 1). Patients’ demographic and disease characteristics were collected. The demographic variables included gender, age, ethnicity, marital status, educational level, occupational type and body mass index (BMI). The characteristics included route of infection, time on EFV-based ART, CD4^+^ T cells counts and viral load.

### Evaluation of generic health

The 12-item Short Form Health Survey (SF-12) is a generic (non-disease specific) rating scale intended to measure perceived physical and mental health and also used as a health-related quality of life measure composed of physical component summary scores (PCS) and mental component summary scores (MCS). We discussed SF-12 from a quality of life perspective in our study. SF-12 is a self-reported measurement and is often used as a health-related quality of life measure. SF-12 total score ranges from 0 to 100, and the higher total score indicates the better perceived quality of life [23].

### Evaluation of anxiety and depression

The Hospital Anxiety and Depression Scale (HADS) comprises 14 items, seven of which relate to symptoms of anxiety (HADS-A) and seven to symptoms of depression (HADS-D). Each item had been answered by the patient on a four-point (0–3) response category and total scores ranged from 0 to 21 for anxiety and depression respectively. The patients with HADS-A or HADS-D scores of > 7 points were regarded as being just suggestive of the presence of anxiety or depression, higher scores indicate worse severity of anxiety or depression [24].

### Evaluation of sleep quality

The Pittsburgh Sleep Quality Index (PSQI) is introduced to assess sleep quality and disturbances. PSQI contains seven individual components: subjective sleep quality, sleep latency, sleep duration, habitual sleep efficiency, sleep disturbances, use of sleeping medication and daytime dysfunction. For each individual components with a range of 0–3, the PSQI global score has a range of 0–21 points. A PSQI score > 5 yielded a diagnosis in distinguishing good and poor sleepers. The higher score indicates the worse sleep quality [25].

### Statistics

All analyses were performed using IBM Statistical Package for the Social Sciences (SPSS Statistics 21.0, SPSS Inc., Chicago, USA). Normality of variable distribution was tested using Kolmogorov–Smirnov test. Normally distributed variables were presented as mean ± standard deviation (SD). Variables with non-normal distributions are shown as medians and interquartile range (IQR), and categorical variables as frequency (percentage). Normally distributed variables were evaluated by one-way analysis of variance (ANOVA) and Kruskal–Wallis test was used for non-normal distributed variables. The chi-square test (or Fisher’s exact test where appropriate) was used for the categorical variables. The correlation analysis was performed using Spearman correlation analysis. Statistical testing was bilateral with statistical significance at *p* < 0.05 and a correlation coefficient > 0.3 was considered to be statistically correlated [26].

## Results

### Baseline characteristics

Four hundred twenty-four PLHIV were enrolled, the mean age was 34 years (IQR, 30–40), 416 (98.1%) were male, and 398 (94.9%) were Han. The mean body mass index (BMI) was 22.8 kg/m ^2^ (IQR, 20.8–24.7). In terms of education levels, 55 (13.0%) patients had a postgraduate degree, 191 (45.0%) had a college degree. 313 (73.8%) were unmarried, 127 (30.0%) were white-collar, and 165 (38.9%) were Blue-collar. Since the population recruited was predominantly male, the most common HIV transmission route was men having sex with men in our study. The average time on EFV-based ART was 3.4 (IQR, 2.0, 5.1) years. The plasma viral load was less than 50 HIV RNA copies/ml in all patients. The mean CD4^+^ T cells counts was 514 cells/μl (IQR, 413–722) and there were significant differences among groups (Table 1).

**Table 1:** Demographic, clinical, and psychosocial characteristics of four groups of PLWH

**Characteristics**	**Total**	**0.5 ≤ ART < 2 year**	**2 ≤ ART < 4 year**	**4 ≤ ART < 6 year**	**ART ≥ 6 year**	***P***
	**(*n=*424)**	**(*n=*106)**	**(*n=*134)**	**(*n=*123)**	**(*n=*61)**	
Sex, n (%)	416 (98.1)	104 (98.1)	132 (98.5)	123 (100)	57 (93.4)	
Age (years), median (IQR)	34 (30-40)	31 (27-36)	33 (30-39)	35 (31-39)	38 (33-47)	<0.001
Ethnicity, n (%)	398/42 (94.9)	100 (94.3)	125 (93.3)	114 (92.7)	59 (96.7)	0.775
Marital status, n (%)						0.058
Unmarried	313 (73.8)	313 (73.8)	79 (74.5)	97 (72.4)	101 (82.1)	
Married	78 (18.4)	78 (18.4)	194 (17.9)	27 (20.1)	16 (13.0)	
Divorced/separated /widowed	33 (7.8)	33 (7.8)	8 (7.5)	10 (7.5)	6 (4.9)	
Education, n (%)						0.421
Postgraduate	55 (13.0)	13 (12.3)	16 (11.9)	20 (16.3)	6 (9.8)	
College degree	191 (45.0)	48 (45.3)	58 (43.3)	60 (48.8)	25 (41.0)	
Technical school graduate	72 (17.0)	14 (13.2)	24 (17.9)	23 (18.7)	11 (18.0)	
below Technical school graduate	106 (25.0)	31 (29.2)	36 (26.9)	20 (16.3)	19 (31.1)	
Employment type, n (%)						0.970
White-collar	127 (30.0)	31 (29.2)	39 (29.1)	40 (32.5)	17 (27.9)	
Blue-collar	165 (38.9)	40 (37.7)	53 (39.6)	49 (39.8)	23 (37.7)	
Student/unemployed /others	132 (31.1)	35 (33.0)	42 (31.3)	34 (27.6)	21 (34.4)	
BMI (Kg/m^2^), median (IQR)	22.8 (20.8-24.7)	22.1 (20.1-24.6)	22.9 (20.8-25.0)	22.9 (20.8-25.0)	22.8 (20.3-24.2)	0.359
Sexual transmission route, n (%)						0.015
Homosexual	304 (71.7)	76 (71.7)	93 (69.4)	99 (80.5)	36 (59.0)	
bisexual	39 (9.2)	6 (5.7)	13 (9.7)	10 (8.1)	10 (16.4)	
Blood borne	9 (2.1)	3 (2.8)	2 (1.5)	0 (0)	4 (6.6)	
others	72 (17.0)	21 (19.8)	26 (19.4)	14 (11.4)	11 (18.0)	
Time on EFV-based ART (years), median (IQR)	3.4 (2.0,5.1)	1.6 (1.2,1.8)	2.9 (2.4,3.3)	4.8 (4.3,5.4)	7.7 (6.8,8.8)	<0.001
CD4^+^ T cells counts (cells/μl), median (IQR)	514 (413-722)	554 (341-652)	601 (401-713)	541 (466-748)	541 (431-708)	0.012
HIV-1 RNA (copies/ml)	<50					

Comparison among four groups was performed by one-way analysis of variance (ANOVA) or Kruskal-Wallis test. *p <* 0.05 was considered statistically significant

*EFV* efavirenz, *ART* antiretroviral therapy, *BMI* Body Mass Index

### The SF-12 scores among different groups were similar

**Fig. 2:**
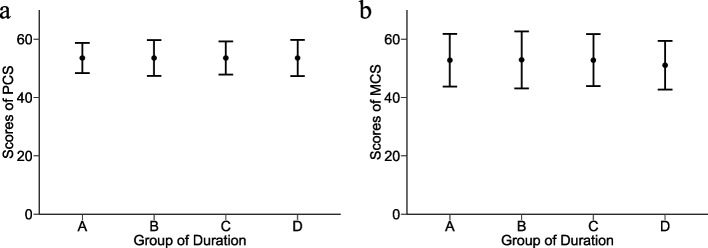
Scores of SF-12 with time on EFV-based ART prolonging. Graphs show the trend of changes in the PCS (**a**) and MCS (**b**) with time on EFV-based ART prolonging. Data are presented as mean (SD). Comparison among four groups was performed by one-way analysis of variance (ANOVA). Abbreviations: *EFV, efavirenz; PCS, physical component summary scores of SF-12; MCS, mental component summary scores of SF-12*

In order to explore differences in generic health among these four groups, SF-12 scores including PCS and MCS were applied. Overall scores of PCS was 51.2 (SD, 6.2), and the PCS scores did not vary significantly among groups (Fig. 2a, p = 0.816). Overall scores of MCS were 50.2 (SD, 9.1). Likely to PCS, there were no significant differences of MCS scores among the groups (Fig. 2b, p = 0.984). PCS and MCS scores had no correlation with age and CD4^+^ T cells counts (Table S1). These results suggested that the SF-12 scores among different groups were similar among different groups with prolongation time on EFV-based ART.

### Prevalence of anxiety and depression did not change with long-term EFV-based ART

**Fig. 3:**
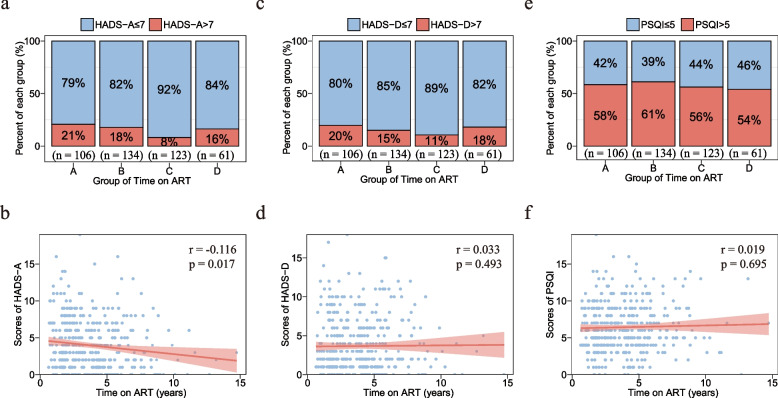
Prevalence of neuropsychiatric adverse events and correlation between score of questionnaires and time on ART. Graphs show the prevalence of anxiety (**a**), depression (**c**) and sleep disturbances (**e**) in each group. Comparison among four groups was performed by chi-square test (or Fisher’s exact test where appropriate). Correlation between scores of HADS-A (b), HADS-D (d) and PSQI (f) and time on EFV-based ART respectively was performed by Spearman correlation analysis. *p* < 0.05 was considered statistically significant and a correlation coefficient > 0.3 was considered to be statistically correlated. Abbreviations: *EFV, efavirenz; ART, antiretroviral therapy; HADS-A, anxiety scores of the Hospital Anxiety and Depression Scale; HADS-D, depression scores of the Hospital Anxiety and Depression Scale; PSQI, scores of the Pittsburgh Sleep Quality Index*

On analysis of HADS-A scores, we found overall prevalence of anxiety (HADS-A scores > 7) was 66 (15.6% of 424 participants) (Fig. 3a). The prevalence of anxiety varied across groups overall (*p* = 0.048), however, pairwise comparisons revealed that all pairwise comparisons among different groups were not significant. In the Spearman correlation analysis, HADS-A scores was weakly correlated with time on EFV-based ART (*r* = -0.116, *p* = 0.017) (Fig. 3b).

On analysis of HADS-D scores, overall prevalence of depression (HADS-D scores > 7) is 65 (15.3%) (Fig. 3c), and no striking differences were observed between groups (*p* = 0.247). In the Spearman correlation analysis, HADS-D scores had no correlation with time on EFV-based ART (*r* = 0.033, *p* = 0.493) (Fig. 3d). HADS-A and HADS-D scores had no correlation with age or CD4^+^ T cells counts, respectively (Table S2). In sensitivity analyses restricted to ≤ 10 and ≤ 8 years on ART, HADS-A scores and HADS-D scores still had no correlation with time on EFV-based ART (Table S2). Based on the HADS-A and HADS-D scores, the frequencies of mild anxiety and depression did not significantly change along with the duration of EFV-based ART similar to generic health.

### Prevalence of sleep disturbances kept high

The PSQI was used to measure sleep disturbances. Prevalence of sleep disturbances (PSQI scores > 5) overall among 424 enrolled patients was 246 (58%), and no differences emerged among the time-on-ART groups (*p* = 0.769) (Fig. 3e). Further, PSQI scores had no correlation with time on EFV-based ART (*r* = 0.019, *p* = 0.695) (Fig. 3f), age or CD4^+^ T cells counts (Table S1). In sensitivity analyses restricted to ≤ 10 and ≤ 8 years on EFV-based ART, PSQI scores had no correlation with time on EFV-based ART (Table S2). In summary, PLHIV have high prevalence of sleep disturbances and sleep disturbances persisted with the extension of time on EFV-based ART.

## Discussion

In the present study, we have shown that the generic mental health, prevalence of anxiety and depression did not significantly change with prolongation of time on EFV-based ART regimen (0.5-15 years) in PLHIV. The prevalence of sleep disturbances was high in PLHIV, and more importantly, it did not change with the duration of ART among those on long-term EFV-based ART. 

According to previous literature, PLWHPLHIV have significantly lower health-related quality of life (HRQoL) than the general population, despite the majority of PLWHPLHIV being virologically and immunologically stable [27]. In our study, by analyzing the scores of SF-12, we found PLWHPLHIV have a lower PCS and MCS scores compared to generalnormal population in China but higher than that of other general chronic illnesses [28]. The results showed that ART-experienced PLWHPLHIV exhibited a high physical and mental health status in China, but there is a gap compared with normal people, at the same time, quality of life didn’t change with the time on ART extending. 

Age and CD4^+^ T cells counts varied across groups in our study. I, in order to confirm that whether age and CD4^+^ T cells counts could influencewere associated with anxiety, depression, and sleep measures, we analyzed the correlation of age and CD4^+^ T cells counts for score of questionnaires. However, there were no correlation between them. PA previous study suggested that psychological and social-demographic factors, rather than HIV disease status, including current CD4^+^T cells counts and viral load were associated with risk of depression and anxiety [29]. The most severe toxicity effects of EFV treatment are consistently reported within the first 2–4 weeks after EFV initiation, and symptoms generally cease after 6–12 weeks [30, 31], however, the patients taking EFV for 3 years were found to have anxiety [32]. In our study, the results of HADS indicated that depression and anxiety persisted in the course of treatment for more than 15 years. Therefore, it is reasonable that analysis of EFV-induced neuropsychiatric adverse events in previous studies was limited by the follow-up durations. As for the prevalence of depression and anxiety, we found higher results (15.6% and 14.615.3`% respectively) compared to that in previous study in 2017 2015 (12.939.6% and 12.849.7% [19] respectively) in China using HADS, however, our results werelower to contemporaneous reports in 2020 (16.9% and 23.0%) according to a meta-analysis [33]. The prevalence of depression and anxiety of this study may have different outcomes from other studies worldwide contemporaneously for different reasons: 1) different scales are used. 2) different female/male ratios. 3) different psychological and social circumstances. However, our results and findings by others may indicated that the prevalence of depression and anxiety were higher than before. There are studies indicated that anxiety and depression are related to COVID-19 [34, 35], and for PLHIV who have more worry and anxiety about the COVID-19 pandemic because of impaired immune response and stigma surrounding if diagnosed with COVID-19, therefore, more provision of confidential care to PLHIV are needed, in consideration of the persistent existence of COVID-19 at present. 

Sleep disturbances are prevalent and distressing symptoms experienced by PLHIV even when their disease is well managed, and it is not only dramatically reducing the quality of life, but also contribute to the progression of disease and non-AIDS comorbidities [20]. Decreased sleep quality is a common side effect among ART-experienced PLHIV, NNRTI in particular [20], and related symptoms mainly occurred in the early stages of treatment [32]. PLHIV taking EFV were observed to have longer sleep latencies and shorter duration of deep sleep in the first weeks of treatment [20], and these symptoms typically resolve 6–8 weeks later [32]. Our study found that PLHIV have high prevalence of sleep disturbances (58%), up to seven fold higher than the general population in 2017 [36] and showed no significant change with the extension of time on EFV-based ART. Further, another study indicated that discontinuation of EFV did not reverse sleep abnormalities [20], however, there are studies have shown that withdrawal from EFV did result in improved self-reported sleep quality among PLWH [37, 38]. Sleep disturbances occur throughout all stages of HIV infection, and may be associated with the virus itself and likelihood of sleep disturbance increases with longer time since infection [39]. Overall, it will be important to assess sleep quality among ART-experienced PLHIV, additionally, PLHIV with sleep disorder should replace the EFV-based ART regimen early. With regard to PLHIV accompanied with neuropsychiatric symptoms, there has some countermeasures. In Chinese Guidelines for Diagnosis and Treatment of HIV/AIDS (2018) [18], a dosage reduction of EFV is suggested to cope with neuropsychiatric adverse events, however, the previous study indicated that the effect of diverse EFV dose to anxiety and depression was not significantly different [40]. According to European AIDS Clinical Society, it would better to replace EFV with another drug for PLHIV with depression. Thus, for the PLHIV with neuropsychiatric adverse events, therapy with the EFV should be stopped and the drug should be replaced. Unfortunately, patients in China are still taking an EFV-based regimen, even with some degree of depression or/and anxiety [19]. Therefore, our results suggest that clinicians of contagion section should pay more attention to neuropsychiatric health. For PLHIV with neuropsychiatric symptoms, depression in particular, clinicians should consider replacing the EFV-based regimen promptly with another ART regimen.

There were several limitations to this study. First, healthy control subjects were not enrolled in the present study thus we cannot separate the effects of HIV and ART. Secondly, as the patients were mainly recruited from a single-center, representative coverage among PLHIV is relatively limited, and improving the area coverage can enhance the accuracy of the analysis findings in further studies. Thirdly, this study was cross-sectional making our comparisons by previous time on ART susceptible to regimen survivorship bias. patients who remained or “survived” on EFV-based ART for many years may be systematically different from patients who have only had the opportunity to remain on ART for a shorter time span. Fourthly, there were relatively low number of women in our study and the participants included were mainly men with a homosexual transmission route, thus our conclusions are only generalizable to men who have sex with men.

## Conclusions

In summary, in ART-experienced PLHIV in China, neuropsychiatric adverse events exist persistently and prevalence did not change with time on EFV-based ART prolonging. The prevalence of sleep disturbances kept high level, suggesting that clinicians should pay more attentions to long-standing psychiatric health and they should perform early and effective intervention, which satisfies clinical requirements to the integrated management of HIV/AIDS in modern society.

## Supplementary Information

**Additional file 1:** **Table S1.** Correlation between age, CD4^+^ T cells counts and score of questionnaires. **Table S2.** Sensitivity analysis restricted to 10 and 8 years on ART for the correlation analysis.

## Data Availability

The datasets used and analysed are stored in a non‑publicly available repository and are available from the corresponding author on reasonable request.
