# Supplementary material for: Anxiety, depression, and sleep disturbances among people on long-term efavirenz-based treatment for HIV: a cross-sectional study in Beijing, China
Source: BMC Psychiatry. 2022 Nov 16;22:710. doi: 10.1186/s12888-022-04366-4 (PMC9667435; doi:10.1186/s12888-022-04366-4)
Supplement: Supplementary file 1 — Additional file 1: Table S1. Correlation between age, CD4+ T cells counts and score of questionnaires. Table S2. Sensitivity analysis restricted to 10 and 8 years on ART for the correlation analysis. [file 12888_2022_4366_MOESM1_ESM.docx]

File name : Additional file

File format: Microsoft Word

Title of data: 1)Table S1. Correlation between age, CD4+ T cells counts and score of questionnaires

2)Table S2. sensitivity analysis restricted to 10 and 8 years on ART for the correlation analysis

Description of data: 1) Table S1: Correlation was performed by Spearman correlation analysis. P < 0.05 was considered statistically significant and a correlation coefficient >0.3 was considered to be statistically correlated. Abbreviations: PCS, physical component summary scores of SF-12; MCS, mental component summary scores of SF-12; HADS-A, anxiety scores of the Hospital Anxiety and Depression Scale; HADS-D, depression scores of the Hospital Anxiety and Depression Scale; PSQI, scores of the Pittsburgh Sleep Quality Index. 2) Table S2: Correlation was performed by Spearman correlation analysis. p < 0.05 was considered statistically significant and a correlation coefficient >0.3 was considered to be statistically correlated. Abbreviations: EFV, efavirenz; HADS-A, anxiety scores of the Hospital Anxiety and Depression Scale; HADS-D, depression scores of the Hospital Anxiety and Depression Scale; PSQI, scores of the Pittsburgh Sleep Quality Index.

| Table S1. Correlation between age, CD4^+^ T cells counts and score of questionnaires | | | | | | | | | | | | | | |
| --- | --- | --- | --- | --- | --- | --- | --- | --- | --- | --- | --- | --- | --- | --- |
|  | PCS | |  | MCS | |  | HADS-A | |  | HADS-D | |  | PSQI | |
|  | R | P |  | R | P |  | R | P |  | R | P |  | R | P |
| Age (years) | 0.07 | 0.14 |  | 0.1 | 0.03 |  | -0.16 | 0.0008 |  | -0.05 | 0.3 |  | -0.06 | 0.23 |
| CD4^+^ T cell counts (cells/μl) | 0.15 | 0.003 |  | 0.03 | 0.49 |  | -0.05 | 0.34 |  | -0.06 | 0.23 |  | -0.06 | 0.27 |

Correlation was performed by Spearman correlation analysis. p < 0.05 was considered statistically significant and a correlation coefficient >0.3 was considered to be statistically correlated. Abbreviations: *PCS, physical component summary scores of SF-12; MCS, mental component summary scores of SF-12; HADS-A, anxiety scores of the Hospital Anxiety and Depression Scale; HADS-D, depression scores of the Hospital Anxiety and Depression Scale; PSQI, scores of the Pittsburgh Sleep Quality Index.*

| Table S2. Sensitivity analysis restricted to 10 and 8 years on ART for the correlation analysis | | | | | | | | |
| --- | --- | --- | --- | --- | --- | --- | --- | --- |
| Time on EFV-based ART (years) | HADS-A | |  | HADS-D | |  | PSQI | |
|  | R | P |  | R | P |  | R | P |
| ≤ 10 years | -0.1 | 0.04 |  | 0.05 | 0.31 |  | 0.04 | 0.46 |
| ≤ 8 years | -0.08 | 0.1 |  | 0.06 | 0.22 |  | 0.01 | 0.77 |

Correlation was performed by Spearman correlation analysis. p < 0.05 was considered statistically significant and a correlation coefficient >0.3 was considered to be statistically correlated. Abbreviations: EFV, efavirenz; *HADS-A, anxiety scores of the Hospital Anxiety and Depression Scale; HADS-D, depression scores of the Hospital Anxiety and Depression Scale; PSQI, scores of the Pittsburgh Sleep Quality Index.*
